# A Disease Modification Effect of APOE *E*4 on the Association between Urinary Albumin Excretion and Cognition in Korean Adults

**DOI:** 10.1155/2014/724281

**Published:** 2014-10-30

**Authors:** Min-Ho Shin, Sun-Seog Kweon, Jin-Su Choi, Young-Hoon Lee, Hae-Sung Nam, Kyeong-Soo Park, Hee Nam Kim, Sun-Young Oh, Seul-Ki Jeong

**Affiliations:** ^1^Department of Preventive Medicine, Chonnam National University Medical School, Gwangju, Republic of Korea; ^2^Department of Preventive Medicine & Institute of Wonkwang Medical Science, Wonkwang University College of Medicine, Iksan, Jeonbuk, Republic of Korea; ^3^Department of Preventive Medicine, Chungnam National University College of Medicine, Daejeon, Republic of Korea; ^4^Department of Preventive Medicine, Seonam University College of Medicine, Namwon, Jeonbuk, Republic of Korea; ^5^Center for Creative Biomedical Scientists, Chonnam National University, Gwangju, Republic of Korea; ^6^Department of Neurology & Research Institute of Clinical Medicine, Chonbuk National University, Biomedical Research Institute of Chonbuk National University Hospital, San 2-20, Geumam-dong, Deokjin-gu, Jeonju, Jeonbuk 561-180, Republic of Korea

## Abstract

*Background*. No previous study examined a disease modifying effect of APOE *E*4 status on the association between the urinary albumin-to-creatinine ratio (UACR) and cognition. This study aimed to investigate whether APOE *E*4 modified the association in Korean adults. *Methods*. We performed a cross-sectional study in adults aged 45 to 74 who were living in Namwon City, Republic of Korea. Cognitive function was measured with the Korean version of modified Mini-Mental State Examination (K-mMMSE) and cognitive impairment was defined as scores falling below the 25th percentile of the K-mMMSE according to age, sex, and educational attainments. *Results*. A total of 10,190 participants (4006 men and 6184 women) were analyzed in the present study. Of these, 1698 subjects (16.7%) were APOE *E*4 carriers. The UACR values were negatively associated with the K-mMMSE scores, even after adjusting for potential confounders including age, sex, education, and vascular risk factors. APOE *E*4 modified the association significantly, resulting in a steeper decline of cognitive function with the increase in UACR in *E*4 carriers (*P* for interaction = 0.021). *Conclusion*. Higher UACR values were significantly associated with cognitive dysfunction in the general Korean population, with cognition in APOE *E*4 carriers being more severely affected by increased UACR.

## 1. Introduction

Cognitive dysfunction and dementia are increasing globally [1]. This increasing trend is particularly problematic in developing countries [2]. Early diagnosis and appropriate intervention are helpful in reducing the socioeconomic burden of the disease. Biomarkers of genetics, neuroimaging, and cerebrospinal fluid, as well as neuropsychological screening examinations, are currently used to understand the exact pathophysiology of the disease and to diagnose it prior to the onset of clinical symptoms. Although there is no strong evidence that vascular interventions prevent dementia, dementia is known to be influenced by vascular diseases or risk factors. Consequently, the ability to measure a representative marker of vascular risk for dementia would be a helpful clinical decision making tool.

The renal arteries share many features with the cerebral arteries, including low resistance, high blood flow, and microvascular autoregulation. Elevations in albumin excretion, or albuminuria, are indicative of renal injury [3]. In addition to renal injury, albuminuria measured via the urinary albumin-to-creatinine ratio (UACR) has become a biologic marker of endothelial dysfunction and underlying systemic diseases such as atherosclerosis [4]. Albuminuria is predictive of the development of type 2 diabetes [5], hypertension [6], and even all-cause mortality in the general population [7].

Albuminuria has also been reported to be associated with cognitive dysfunction [8], cognitive decline [9], and dementia [10]. However, most of studies have been performed in Caucasian populations, with no previous studies having been performed among Asian populations. In the present study, we examined (A) the association between UACR and cognitive performance in Korean adults and (B) a disease modifying effect of APOE* E*4, a well-known genetic risk marker of dementia, on this association. The APOE* E*4 effect on dementia has been reported to be stronger in Asian than in other ethnicities [11]. Using the data set from the Namwon study, all possible confounders including age, sex, educational attainment, vascular risk factors, kidney markers, and depression scale were included in the present analysis. With respect to cognitive scores, cognitive impairment was defined as a score which falls below the 25th percentile according to age, sex, and educational attainments.

## 2. Subjects and Methods

### 2.1. Study Population

The Namwon study is an ongoing prospective study designed to investigate the prevalence, incidence, and risk factors for chronic diseases in a rural population. Details of the study participants and measurements have been published previously [12]. A total of 10,667 participants (4,201 men and 6,466 women) were recruited in the baseline survey between January 2004 and February 2007 in Namwon city of Jeonbuk province in South Korea. Of these participants, 477 were excluded because of missing cognition, education, UACR, or APOE data. Following these exclusions, data on 10,190 participants (95.5%; 4,006 men, 6,184 women) were included in the present analyses. This study was conducted in accordance with the Declaration of Helsinki guidelines. The study protocol was independently approved by the Institutional Review Boards of Chonnam National University Hospital and Chonbuk National University Hospital, and informed consent was obtained from each participant.

### 2.2. APOE Genotyping

Genomic DNA was extracted from peripheral blood with an AccuPrep Genomic DNA Extraction Kit (Bioneer, Seoul, Korea) or a QIAamp DNA Mini Kit (Qiagen Inc., Chatsworth, CA, USA) according to the manufacturers' protocol. APOE genotypes were determined as described by Hixson and Vernier, with slight modifications [13]. Our APOE genotyping method has been reported previously [14].

### 2.3. Measurement of Cognitive Function

An apparently healthy population with no limitation of motion and no behavioral problems was screened for cognitive function, as described previously [14]. Cognitive function was measured using the Korean version of the modified Mini-Mental State Examination (K-mMMSE), which was validated and proven to result in a finer discrimination of cognitive impairment and dementia in a Korean elderly population [15]. The K-mMMSE is scored on a 100-point scale with higher scores indicating greater cognition. From the global cognitive measures, scores of individual cognitive domains including attention (digit span backward), orientation (time and space), memory (registration, immediate and delayed recall, and remote memory), language (reading, writing, naming, and repetition), frontal executive function (word fluency, abstract reasoning, and 3 step execution), and visuospatial functions (interlocking pentagon) were extracted and used for statistical analyses. A project neurologist (SKJ) supervised the examinations, and two additional neurologists independently administered the final scoring of the K-mMMSE. Cognitive impairment was defined as scoring below the 25th percentile according to age, sex, and educational attainments.

### 2.4. Assessments and Measurements

The clinical data collected from the subjects included prior history of cardiovascular disease (CVD), CVD risk factors, and medication usage. Major CVD risk factors included hypertension (blood pressure ≥ 140/90 mmHg or use of antihypertensive medication), type 2 diabetes (current use of glucose-lowering medications or fasting blood glucose > 7.0 mmol/L (126 mg/dL) or hemoglobin A1c ≥ 6.5%), and hyperlipidemia (current use of lipid-lowering medications, total cholesterol ≥ 6.2 mmol/L (240 mg/dL), or low density lipoprotein (LDL) cholesterol ≥ 4.1 mmol/L (160 mg/dL)). Smoking status was divided into current and noncurrent smokers (former smokers and nonsmokers). Participants also answered questions concerning alcohol consumption and were then divided into current or noncurrent drinkers. Body mass index (BMI) was calculated as weight divided by height squared (kg/m^2^). Blood pressure was measured on the right arm with a standard mercury sphygmomanometer after a 5 min rest in a sitting position. Blood pressure readings were recorded to the nearest even number, and three readings were taken at 1 min intervals from each participant. The mean value of the three readings was considered to represent the individual's blood pressure. Depressed mood was assessed with the Center for Epidemiological Study-Depression Scale Korean version (CES-DK). Higher scores of CES-DK indicate greater depressed mood, a finding validated in a Korean population [16].

Blood samples were drawn after a 12-hour overnight fasting period and standard techniques were used to measure serum chemistries and lipids. Each participant provided an early morning spot urine sample in a sterile container at the examination center. Urinary albumin concentrations were measured using a turbidimetric immunoassay, and creatinine concentrations were measured using the Jaffe method on an automated analyzer (Hitachi-7600, Hitachi, Ltd.). All laboratory procedures were conducted at the same laboratory. The UACR was defined as the urinary albumin value divided by the urinary creatinine concentration (mg/g). Kidney function was assessed using an estimated glomerular filtration rate (eGFR, mL/min/1.73 m^2^), which was calculated using the MDRD formula as follows [17]: 186.3 × (serum creatinine)^−1.154^  × (age)^−0.203^  × 0.742 [if female], with the serum creatinine concentration expressed in mg/dL.

## 3. Statistical Analysis

Data are expressed as mean ± standard deviation (SD) or percentages. Student's *t*-tests were used to analyze differences between continuous variables needs, while chi-square tests were used for categorical variables. Because the effect of the UACR differed by APOE status, an APOE* E*4-stratified analysis was performed. Participants were categorized by UACR scores into the following groups: <15, 15–29, 30–299, and ≥300 mg/g. Analysis of covariance (ANCOVA) was used to evaluate the association between UACR categories and cognitive function. A logistic regression model was used to evaluate the association between the UACR categories and cognitive impairment. In the multivariate models, age, sex, educational levels, BMI, eGFR, CES-DK scores, hypertension, type 2 diabetes, hyperlipidemia, and smoking and drinking status were adjusted. Interactions terms like APOE* E*4 status *Χ* UACR categories in each cognitive domain were created, and their significance was assessed. Hardy-Weinberg equilibrium was tested by use of a chi-square goodness-of-fit test. All statistical analyses were conducted using PASW statistics 18 (SPSS Inc., Chicago, IL, USA). Statistical significance was set at *P* < 0.05.

## 4. Results

The mean (±SD) and interquartile ranges of the K-mMMSE scores according to age, sex, and educational attainments are described in Table 1. The K-mMMSE scores were significantly increased in younger and more highly educated participants (*P* < 0.001). The other demographics at baseline are presented in Table 2. 1698 participants (16.7%) were APOE* E*4 positive and grouped as APOE* E*4 carriers. Within these participants, 107 (6.3%) were APOE* E *2/4, 1503 (88.5%) were* E* 3/4, and 88 (5.2%) were* E *4/4. In accord with APOE* E*4 status, hyperlipidemia and eGFR were significantly higher in the* E*4 carriers. Otherwise, there was no significant difference with respect to APOE* E*4 status.

The UACR values were negatively and independently associated with the K-mMMSE scores in both APOE* E*4 carriers and noncarriers, even after adjusting for potential confounders, as shown in Tables 3 and 4. Although the associations were significant in both carriers and noncarriers, there was a significant interaction between UACR and APOE* E*4 status on cognitive function (*P* for interaction = 0.021). In the APOE* E*4 carriers, the slope of the decline was steeper, especially in subjects with macroalbuminuria (UACR ≥ 300 mg/g). The variance of the K-mMMSE scores explained by the UACR after adjustment for covariates was 1.6% and 0.6% in the APOE* E*4 carriers and noncarriers, respectively.

All the cognitive domains, except visuospatial function, showed significant and independent associations with UACR in APOE* E*4 noncarriers, even after adjusting for possible confounders, as shown in Tables 3 and 4. In the APOE* E*4 carriers, significant differences were observed in memory, orientation, visuospatial function, and language. Among the cognitive domains, the variance of frontoexecutive function explained by the UACR categories in APOE* E*4 noncarriers was 0.5% and that of memory in the APOE* E*4 carriers was 2.2%. With respect to the various domains, the memory and visuospatial function domains showed significant interaction terms, and the orientation domain reached significance (*P* = 0.052).

Higher values of UACR showed significant and independent associations with cognitive impairment, as shown in Table 5. In the APOE* E*4 noncarriers, odds ratio (OR) of the UACR values ≥300 mg/g (macroalbuminuria) for cognitive impairment was 1.87 (95% CI, 1.29–2.72) compared to UACR values of <15 mg/g, while in the APOE* E*4 carriers, OR was 3.56 (1.34–9.42). Increasing UACR scores showed a significant linear trend with respect to cognitive impairment in both groups.

## 5. Discussion 

The present study, performed in a Korean adult population, showed that increased UACR was significantly associated with lower cognitive function. Furthermore, APOE* E*4 status modified this effect of UACR on cognitive function. Compared to the APOE* E*4 noncarriers, the APOE* E*4 carriers showed a steeper cognitive decline. This was particularly evident in subjects with macroalbuminuria. Among the cognitive domains, memory and visuospatial function also showed significant interactions. To the best of our knowledge, this is the first report to show an interactive effect of APOE* E*4 on the association between UACR and cognitive function. All previous studies examining the associations between urinary albumin excretion and cognitive performance [18, 19], cognitive decline [20, 21], or dementia [10] have been performed in Caucasian populations, although some studies did include African Americans [10]. The present study is also the first to show a significant association between UACR and cognitive performance in an Asian population.

APOE* E*4 status significantly modified the association between UACR and cognitive function. The declining slope was steeper among APOE* E*4 carriers than* E*4 noncarriers. The effects of the APOE genotype on the increased risk of Alzheimer's disease are likely to be mediated by the differential effects of apoE4 on amyloid-*β* accumulation in the brain [22, 23]. In addition to increased cerebral deposition [24], amyloid-*β* is known to accumulate within the perivascular channels around capillaries and arteries and to cause failure of drainage of interstitial fluid in white matter [25]. Increased urinary albumin excretion is also significantly associated with small vessel disease [26] or white matter hyperintensities [27]. Among the cognitive domains, frontal executive dysfunction, a representative marker of small vessel disease, has been most commonly reported to be associated with increased urinary albumin excretion [8, 18, 19, 28]. In the present study,* E*4 status did not show a significant interaction with frontal executive function. By contrast,* E*4 status showed significant interactions with memory and visuospatial functions. Memory and visuospatial function are cognitive manifestations of temporal and occipital lobes. This suggests that APOE* E*4 and increased urine albumin excretion have synergistic adverse effects on cortical and subcortical cognitive functions, respectively.

The renal arteries share common features with the cerebral arteries, including low resistance, high blood flow, and microvascular autoregulation. As the kidneys and brain share a high demand for energy and a lack of fuel reserves, these common features of the renal and cerebral arteries are likely a common defense mechanism [29]. This suggests that both albuminuria and cognitive impairment share similar underlying vascular pathologic processes. UACR cannot be considered a direct measure of cognitive reserve, unlike the use of educational attainment as a measure of potential intellectual capacity [30] or of arm length as a measure of childhood growth [31]. Rather, UACR serves as a measure of current vascular status, such as endothelial dysfunction or atherosclerosis. UACR can be modified with appropriate interventions including physical exercise, consumption of a low protein diet, and the appropriate use of antihypertensives [32]. In a previous randomized controlled trial, control of UACR with angiotensin converting enzyme inhibitor or angiotensin II receptor blockade was associated with a decrease in cognitive decline [9]. Further studies will be needed to determine whether these beneficial effects would be greater in APOE* E*4 carriers.

In the present study, the APOE* E*4 carriers with microalbuminuria (UACR 30–299 mg/g) did not show significant differences in cognitive function compared to those with normal UACR values. This was not due to differences in age or current medications, including antihypertensives or antidiabetics (data not shown). In our cohort, women were significantly more likely than men to present with microalbuminuria (UACR 30–299 mg/g) [33]. However, we could not delineate further whether this discrepancy was due to the sex effect on microalbuminuria.

There are several notable limitations of our study. First, due to the cross-sectional study design, a causal relationship between UACR values and cognition cannot be inferred. In addition to a longitudinal study, fundamental mechanisms for the parallel dysfunction of the two remote organs should be sought. Second, a single measurement of UACR may not adequately characterize the* in vivo* variability of this value. Although we adjusted for urinary creatinine excretion, the assessment of albumin excretion with spot urine tests may be less accurate than 24-hour urine collections or first morning voids. Third, cognitive function was measured with the global measure of K-mMMSE and cognitive scores of the various domains were extracted from the entire battery. The extracted score of each cognitive domain was diverse and limited, especially with respect to range (e.g., maximum score was 26 for memory, but 7 for attention). The small range of some cognitive domains might undermine statistical power, thereby hindering the ability to reveal true associations between UACR and cognitive domains.

## 6. Conclusion 

In Korean adults, UACR values were independently and negatively associated with cognitive performance, and APOE* E*4 carriers showed a steeper cognitive decline compared to* E*4 noncarriers. Among the cognitive domains evaluated, memory and visuospatial function also showed significant interactions. Further studies are needed to evaluate the generalizability of the present findings.

## Figures and Tables

**Table 1:** Distributions of K-mMMSE and cutoff values for cognitive impairment according to age, sex, and formal educational attainments.

	Men			Women			*P*
	No	Mean ± SD	25, median, 75% tile	No	Mean ± SD	25, median, 75% tile	
Age, 45–54 yrs							
Formal education: none	32	71.7 ± 17.3	69, 74, 84	188	69.3 ± 15.7	63, 72, 79	0.437
1–6 yrs	305	84.9 ± 9.7	80, 87, 92	739	82.6 ± 9.5	78, 84, 89	0.001
More than 6 yrs	449	90.8 ± 7.6^*^	88, 92, 96	578	90.4 ± 6.7^*^	87, 92, 95	0.332
Irrespective of education	786	87.7 ± 9.9	84, 90, 94	1505	83.9 ± 11.6	79, 86, 92	<0.001
Age, 55–64 yrs							
Formal education: none	195	71.3 ± 14.1	64, 73, 82	1045	66.0 ± 12.7	58, 67, 74	<0.001
1–6 yrs	709	82.3 ± 9.7	77, 84, 89	1025	80.1 ± 10.5	75, 81, 88	<0.001
More than 6 yrs	605	88.4 ± 7.5^*^	84, 90, 94	288	87.5 ± 7.8^*^	83, 89, 94	0.098
Irrespective of education	1509	83.3 ± 11.0	78, 85, 91	2358	74.7 ± 13.9	66, 77, 85	<0.001
Age, 65–74 yrs							
Formal education: none	364	70.8 ± 12.4	64, 72, 80	1717	60.8 ± 13.7	52, 62, 70	<0.001
1–6 yrs	806	80.2 ± 10.7	75, 82, 87	521	75.9 ± 11.5	70, 78, 84	<0.001
More than 6 yrs	541	86.8 ± 8.1^*^	82, 88, 93	83	82.5 ± 9.8^*^	76, 85, 89	<0.001
Irrespective of education	1711	80.3 ± 11.9	74, 82, 89	2321	65.0 ± 14.9	55, 66, 76	<0.001

*P* values by independent *t*-tests comparing men and women.

^*^ *P* < 0.001 by analysis of variance (ANOVA) according to educational attainments.

**Table 2:** Demographics of participants according to APOE *E*4 status.

	*E*4 noncarrier	*E*4 carrier	*P*
Number (%)	8492 (83.3%)	1698 (16.7%)	
Age, y	61.6 ± 7.8	61.5 ± 7.7	0.494
Women, %	60.4	62.0	0.220
Formal education, (%)			
None	34.8	34.5	0.839
1~6 years	40.3	40.4	
More than 9 years	24.9	25.1	
CES-DK	52.1 ± 8.6	52.5 ± 8.4	0.139
Current smoker, %	15.4	14.7	0.432
Current drinker, %	47.6	46.4	0.369
Hypertension, %	40.0	39.3	0.574
Type 2 diabetes, %	13.5	13.1	0.656
Hyperlipidemia, %	10.2	13.0	0.001
Body mass index, kg/m^2^	24.4 ± 3.1	24.4 ± 3.1	0.756
Systolic blood pressure, mmHg	126.1 ± 18.3	125.7 ± 18.4	0.415
Diastolic blood pressure, mmHg	80.5 ± 10.2	80.3 ± 10.1	0.543
Fasting blood glucose, mg/dL	104.9 ± 24.0	104.0 ± 23.4	0.148
UACR, mg/g	44.7 ± 146.7	43.0 ± 161.6	0.654
eGFR^*^, *µ*mol/L	77.7 ± 14.6	78.4 ± 14.5	0.049

Data are expressed as mean ± SD or percentages. CES-D-K: the Center for Epidemiological Study-Depression Scale Korean version, UACR: urinary albumin-to-creatinine ratio, and eGFR: estimated glomerular filtration rate.

^*^eGFR was calculated with the 4-variable modification of diet in renal disease (MDRD) formula.

*P* values are shown for chi-square tests and *t*-tests, as appropriate.

**Table 3:** Adjusted mean (±SE) scores of K-mMMSE and cognitive domains according to categories of UACR in APOE *E*4 noncarriers.

	K-mMMSE and cognitive domain scores according to UACR (mg/g)				*P* for overall	*P* for linear	Partial eta-squared, %
	<15.0	15.0–29.9	30.0–299.9	≥300.0	difference	trend	
Number	4147	2169	1988	188			
Global							
K-mMMSE (100)	78.7 ± 0.2	77.6 ± 0.3	76.9 ± 0.3	74.9 ± 0.9	<0.001	<0.001	0.6
Domains							
Attention (7)	3.8 ± 0.0	3.7 ± 0.0	3.6 ± 0.0	3.3 ± 0.1	<0.001	<0.001	0.3
Memory (26)	20.9 ± 0.1	20.6 ± 0.1	20.6 ± 0.1	19.9 ± 0.3	0.005	0.003	0.2
Orientation (20)	18.6 ± 0.0	18.5 ± 0.1	18.2 ± 0.1	18.1 ± 0.2	0.003	<0.001	0.3
Visuospatial (10)	8.2 ± 0.0	8.1 ± 0.0	8.0 ± 0.1	7.9 ± 0.2	0.053	0.006	0.1
Language (18)	15.2 ± 0.0	15.1 ± 0.1	14.9 ± 0.1	14.6 ± 0.2	<0.001	<0.001	0.3
Frontoexecutive (19)	11.9 ± 0.1	11.6 ± 0.1	11.5 ± 0.1	11.1 ± 0.3	<0.001	<0.001	0.5

Data are expressed as mean ± standard error. The numbers in the brackets following domain names represent the total (maximum) score.

Adjusted for age, sex, educational attainments, smoking status, alcohol use, BMI, eGFR, hypertension, type 2 diabetes, hyperlipidemia, and CES-DK.

**Table 4:** Adjusted mean (±SE) scores of K-mMMSE and cognitive domains according to categories of UACR in APOE *E*4 carriers.

	K-mMMSE and cognitive domain scores according to UACR (mg/g)				*P* for overall	*P* for linear	Partial eta-squared, %
	<15.0	15.0–29.9	30.0–299.9	≥300.0	difference	trend	
Number	852	402	421	23			
Global							
K-mMMSE (100)	78.2 ± 0.4	75.3 ± 0.6	77.0 ± 0.6	70.0 ± 2.6	<0.001	0.007	1.6
Domains							
Attention (7)	3.6 ± 0.1	3.5 ± 0.1	3.7 ± 0.1	3.0 ± 0.4	0.236	0.891	0.3
Memory (26)	21.2 ± 0.2	19.5 ± 0.2	20.5 ± 0.2	19.4 ± 1.0	<0.001	0.003	2.2
Orientation (20)	18.6 ± 0.1	18.2 ± 0.2	18.2 ± 0.2	16.3 ± 0.7	0.002	0.003	1.1
Visuospatial (10)	8.1 ± 0.1	7.8 ± 0.1	8.2 ± 0.1	7.3 ± 0.5	0.022	0.823	0.7
Language (18)	15.1 ± 0.1	15.0 ± 0.1	14.9 ± 0.1	13.4 ± 0.6	0.029	0.079	0.7
Frontoexecutive (19)	11.8 ± 0.1	11.5 ± 0.2	11.5 ± 0.2	10.2 ± 0.7	0.107	0.083	0.5

Data are expressed as mean ± standard error. The numbers in the brackets following domain names represent the total (maximum) score.

Adjusted for age, sex, educational attainments, smoking status, alcohol use, BMI, eGFR, hypertension, type 2 diabetes, hyperlipidemia, and CES-DK.

**Table 5:** Association between cognitive impairment^*^ and categories of UACR.

Categories of UACR, mg/g	APOE *E*4 noncarrier		APOE* E*4 carrier	
<15.0	1.00		1.00	
15.0–29.9	1.16 (1.00–1.34)	0.052	1.64 (1.19–2.26)	0.002
30.0–299.9	1.31 (1.12–1.52)	0.001	1.26 (0.90–1.76)	0.172
≥300.0	1.87 (1.29–2.72)	0.001	3.56 (1.34–9.42)	0.011
*P* for trend		<0.001		0.024

Odds ratio and 95% confidence intervals are presented.

Adjusted for the same covariates as in Table 2.

^*^Cognitive impairment was defined as K-mMMSE lower than 25th percentile according to age, sex, and educational attainments.
